# Impact of physical activity and psychological skills training with parental participation on cognitive, emotional, and behavioral outcomes in children with ADHD and their parents: A study protocol

**DOI:** 10.1371/journal.pone.0325669

**Published:** 2025-06-05

**Authors:** Bumcheol Kim, Ejin Park, Jaewon Kim

**Affiliations:** 1 Department of Physical Medicine and Rehabilitation, College of Medicine, The Catholic University of Korea, Seoul, Republic of Korea; 2 Department of Psychiatry, Incheon St. Mary’s Hospital, College of Medicine, The Catholic University of Korea, Seoul, Republic of Korea; 3 Department of Physical Medicine and Rehabilitation, Incheon St. Mary’s Hospital, College of Medicine, The Catholic University of Korea, Seoul, Republic of Korea; University of Montenegro, MONTENEGRO

## Abstract

**Background:**

Attention Deficit/Hyperactivity Disorder (ADHD) is a neurodevelopmental disorder characterized by difficulties in attention, hyperactivity, and impulse control. Previous studies have suggested that engaging in moderate to vigorous physical activity (PA) and undergoing psychological therapy can help improve ADHD symptoms when combined with pharmacological treatments. This study aims to investigate the effects of parent participation in physical activities and psychological skills training (PST) on the cognitive, emotional, and behavioral outcomes of children with ADHD, as well as the emotional well-being of their parents.

**Methods:**

Participants for the study will be recruited from a tertiary medical institution. The focus will be on children aged 6–12 years who have been diagnosed with ADHD and are currently receiving treatment at the hospital. Assessments will be conducted separately for the children and their parents. We will evaluate the children’s cognitive, emotional, and behavioral aspects, while for the parents, we will assess only emotional and behavioral aspects.

The study will consist of three Intervention Groups (IG 1 and IG 2) and one control group. IG 1 will receive a combined intervention of PA and PST, IG 2 will participate in PA alone, and the control group will not receive any intervention.

**Discussion:**

This study’s findings underscore the benefits of greater active participation when children participate in activities with their parents. It will also demonstrate the possible advantages of integrating PA with PST. The research will also explore how these interventions may influence parents, providing insights into whether their engagement enhances their psychological well-being.

**Trial registration:**

This study is registered at Clinical Research Information Service (CRIS, https://cris.nih.go.kr) as KCT0010046

## Introduction

Attention Deficit/Hyperactivity Disorder (ADHD) is a neurodevelopmental disorder characterized by a short attention span, inattention, hyperactivity, and difficulties with impulse control [1]. Pharmacotherapy has shown effective results in the short term and is primarily recommended as the first-line treatment option [2]. Most guidelines recommend prioritizing psychoeducation and behavioral management as the first treatment options unless symptoms and impairments are especially severe. There is a general agreement that behavioral management, mainly through parent training, is preferred for children under six [2,3].

Previous studies have investigated interventions incorporating physical activity (PA), medication, and behavioral management [4,5]. These studies have shown that moderate to vigorous PA significantly enhances the neurophysiological, cognitive, emotional, and behavioral aspects of adolescents with ADHD while improving attention, cognitive and executive function, and motor skills [5–7]. Additionally, PA decreases symptoms of depression and anxiety, enabling individuals to manage stress more effectively [8,9]. PA can enhance biological brain health by improving several neurotransmitter systems connected to the hippocampus, upregulating brain-derived neurotrophic factor, and promoting neurogenesis [10,11]. Releasing dopamine, serotonin, and norepinephrine neurotransmitters can enhance attention and memory [12,13].

Psychological skills training (PST) is a technique aimed at developing the ability to manage one’s psychological state positively. This type of training is beneficial not only for athletes but also for individuals in various performance-based fields, such as doctors, soldiers, and performing artists. It helps them achieve peak performance [14–16]. The training includes goal setting, establishing routines, visualization techniques, concentration exercises, anxiety management, mindfulness practices, and positive self-affirmation. When applied to children with ADHD and their parents, this training has demonstrated a decrease in parenting stress and anxiety while also effectively enhancing cognitive, emotional, and behavioral aspects like self-confidence and attention [14,17,18]. Previous studies have indicated that intervention programs for children with ADHD and their parents may contribute to positive enhancements in their emotional and behavioral functioning.

The mental health of parents greatly influences not just their well-being but also their parenting practices as well as the development and mental well-being of their children [19,20]. Parents of children with ADHD experience more parenting stress than parents of children without clinical diagnoses. Additionally, the severity of ADHD symptoms correlates with increased parenting stress, creating a vicious cycle between the two [21,22]. This indicates that interventions should focus on both the children and their parents when addressing ADHD. Moreover, the impact of the interventions may be more significant when both the child and the parents participate in treatments [23]. Parents raising children with disabilities often encounter various challenges that hinder their ability to engage in independent activities away from their children, resulting in difficulties in consistently participating in intervention programs [24,25].

Considering this context, the goal of this study is to explore the impact of PA and PST carried out in tandem by children with ADHD and their primary caregivers. We will assess the effects on the children’s cognitive abilities, emotional states, and behavior. Furthermore, we will investigate how these interventions affect the caregivers’ emotional well-being and anxiety levels. By involving children and their caregivers in these interventions, this study seeks to improve the children’s engagement in PA and PST while providing support to the caregivers to enhance the overall outcomes.

## Materials and methods

### Study design

This prospective randomized controlled intervention received approval from the Institutional Review Board of Incheon St. Mary’s Hospital (OC24FISI0032) on April 22, 2024. The protocol complies with the Helsinki Declaration and Good Clinical Practice guidelines. Participants will be divided into three groups: intervention group 1 (PA and PST), intervention group 2 (PA), and a control group (no intervention).

Initially, the two intervention groups will be categorized by age. We will randomly assign 18 children and their parents to each group, resulting in 72 participants, using a random number generator (version 1.1). The control group will consist of 18 children and their parents (36 participants) selected from those unable to participate in the interventions but who could complete the assessments. Therefore, the total number of participants across all groups will be 54 children, 54 parents, and 108 individuals.

To enhance the robustness of the analysis and mitigate potential biases from participant dropout, an intention-to-treat (ITT) approach will be implemented. All randomized participants will be analyzed regardless of intervention adherence. Missing data will be managed using multiple imputation methods to preserve data integrity and minimize bias. Additionally, sensitivity analyses will be conducted to evaluate the potential impact of missing data on the study outcomes.

**Fig 1:**
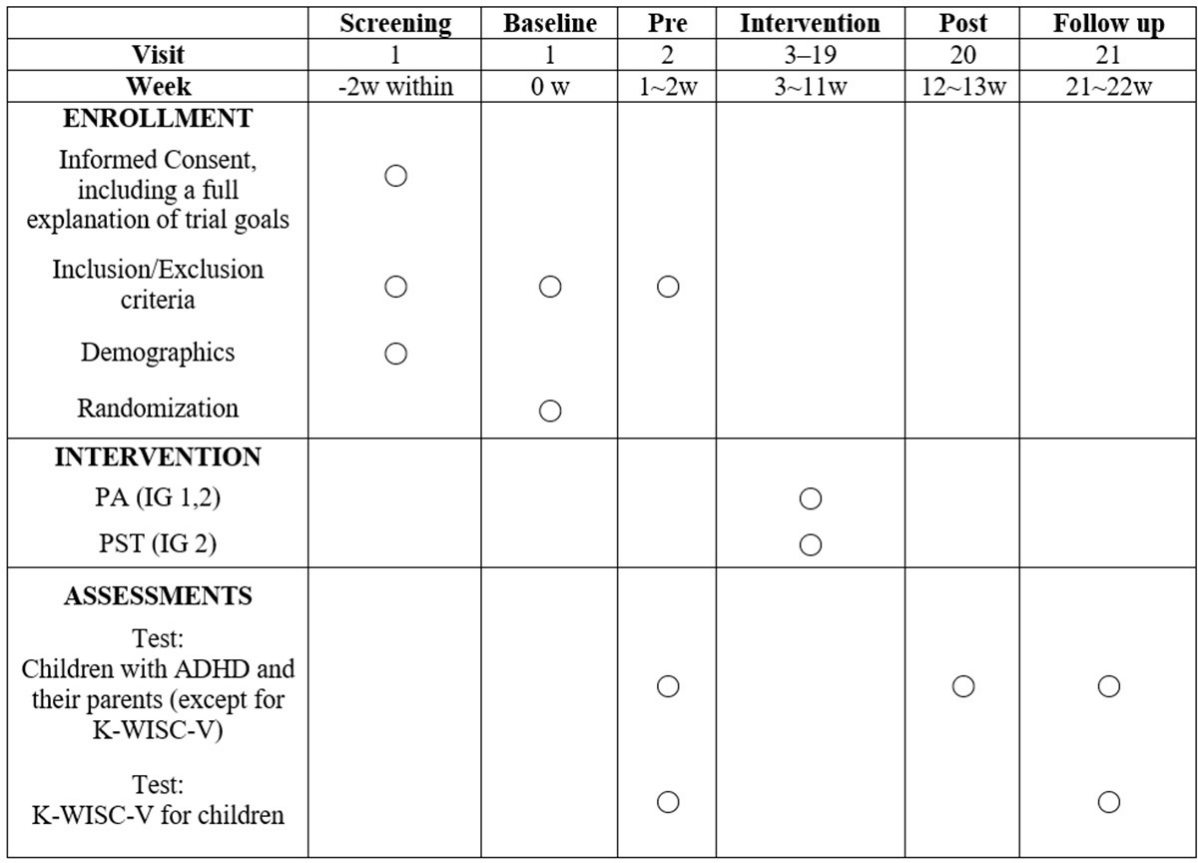
Schedule of enrolment, interventions, and assessments. IG: Intervention Group; CC: Control Group; PA: Physical Activity; PTS: Psychological Skills Training.

All participants in this study will continue to receive their existing medication treatments throughout the intervention period. Before proceeding with the assessments and interventions, the children and their parents will provide written informed consent. Recruitment and data collection began on May 31, 2024 and is expected to end on February 28, 2025. Data collection and results are expected to be completed by September 30, 2025. Details of the research procedure are presented in Fig 1.

### Participants

#### Selection criteria and recruitment.

Participants for this study will be recruited using purposive sampling, a type of non-probability sampling. Eligible participants will be children diagnosed with ADHD by pediatric psychiatrists at local child and adolescent psychiatry clinics and regional tertiary hospitals where the intervention is conducted. Recruitment announcements will be displayed on hospital screen bulletin boards and within the local community. The inclusion criteria for this study are as follows: 1) Children aged 6–12 years who have received a formal diagnosis of ADHD and are currently undergoing treatment; 2) Children for whom previous Wechsler test results confirm the absence of cognitive decline; 3) Children and parents where the primary caregiver is available to participate in the assessment and intervention. The exclusion criteria for this study include: 1) Children with significant cognitive decline; 2) Children with mental, physical, or medical conditions that would impede participation in PA or PST; 3) Children with any co-occurring disabilities other than ADHD; 4) Children whose primary caregiver is unable to participate; 5) Children involved in other research studies or exercise programs; 6) Children who struggle to cooperate with assessments and interventions. Among children diagnosed with ADHD receiving treatment, those who meet the inclusion/exclusion criteria and desire to participate will be selected.

### Sample size

The sample size for this study was determined through statistical analysis using the G*Power 3.1 program. Based on the data analysis method, the test family was set to F-tests, and the statistical test was designated as ANOVA: repeated measures, within-between interaction. The effect size was determined according to Cohen’s method [26] and was set to 0.25, based on previous studies [27–29]. Initially, the significance level (α) was set at 0.05 for the sample size calculation. However, to account for multiple comparisons, the Bonferroni correction was applied, adjusting the per-comparison significance level to α = 0.0167. The statistical power (1-β) was set at 0.80, the number of groups at three, and the number of repeated measurements at three. Consequently, the required sample size was recalculated to be 45 participants (15 per group). To account for potential attrition, a 20% dropout rate was considered, based on rates observed in previous studies [30–32]. Therefore, the final total sample size was 54 participants, with 18 participants allocated to each group.

### Intervention

In this study, intervention group 1 will receive PA and PST, while intervention group 2 will only receive PA. Both groups will engage in the intervention program alongside their primary caregiver. The control group will not receive any intervention. Throughout this process, the child will continue with their existing treatment, and if applicable, the additional intervention will be implemented.

### Physical activity (PA)

The PA intervention program is based on the meta-analysis conducted by Kim et al. (2022) [6]. On the effects of PA in children and adolescents with ADHD. Specifically, we used the PA program (aerobic exercise and manipulative exercises) from the parent-involved intervention study by Kim et al. (2024) [23,33]. The PA program will be conducted over eight weeks, consisting of two weekly sessions. Each session will last for 90 minutes and include 30 minutes of manipulative exercises followed by 60 minutes of aerobic exercise. The intensity of the PA will be maintained at a moderate to vigorous level, as indicated by a modified Borg Scale rating of 5–7. The level of physical activity will be monitored using the ActiGraph wCT3x-BT (ActiGraph, Pensacola, FL, USA), and efforts will be made to maintain the intensity of moderate to vigorous activity. All sessions will occur in a group setting, with primary caregivers and parents participating. Detailed information about the PA program can be found in Table 1.

**Table 1:** Physical activity program.

Week	Type	Program Contents
1	manipulative	Rolling and receiving a ball, pushing and stopping a ball with the foot (1m), bouncing a ball in place, and throwing and receiving a ball (1m)
	Aerobic	Horse running, jumping with both feet, step exercises, 10 m shuttle run (10 repetitions) 2 sessions
2	manipulative	Rolling and receiving a ball, pushing and stopping a ball with the foot (2m), bouncing a ball while walking, and throwing and receiving a ball (1m)
	Aerobic	Alternating jumps, jumping with both feet, step exercises, 10 m shuttle run (11 repetitions), 2 sessions.
3	manipulative	Rolling and receiving a ball, pushing and stopping a ball with the foot (3m), bouncing a ball while walking (zigzag), throwing and receiving (1.5m)
	Aerobic	Horse running, jumping with both feet, step exercises, climbing, 10 m shuttle run (12 repetitions) 2 sessions
4	manipulative	Rolling a ball while moving and receiving, pushing a ball while driving and stopping, bouncing a ball while running, and throwing and receiving (2m)
	Aerobic	Alternating jumps, jumping with both feet, step exercises, and a 10 m shuttle run (13 repetitions) for 2 sessions.
5	manipulative	Rolling a ball while moving and receiving, pushing a ball while driving and stopping, bouncing a ball while crossing a balance beam, and throwing and receiving a ball.
	Aerobic	Jumping with both feet, climbing, sliding, egg laying, 10 m shuttle run (14 repetitions) 2 sessions
6	manipulative	Hitting a stationary ball (ground, T-ball), hitting a rolling ball, hitting a flying ball
	Aerobic	Jumping with both feet, climbing, sliding, egg laying, 10 m shuttle run (15 repetitions) 1 session
7	manipulative	Kicking a stationary ball, kicking a rolling ball, dribbling a ball while moving in a zigzag
	Aerobic	Zigzag running, 10 m shuttle run, single-leg, double-leg, horse, alternating jumps, climbing.
8	manipulative	Solo balloon volleying, returning flying objects, and balloon tennis
	Aerobic	Zigzag running, 10 m shuttle run, single-leg, double-leg, horse, alternating jumps, climbing.

### Psychological Skills Training (PST)

A PST program specifically designed for children with ADHD will be implemented, grounded in essential principles and evidence-based practices of PST. This program will incorporate routines, goal setting, positive self-affirmation, concentration training, and anxiety management techniques (see Table 2). The training aims to enhance attention and alleviate stress and anxiety, which are frequently linked to ADHD. Sessions will be conducted once a week for 50 minutes over 8 weeks, with both primary caregivers and their children participating together in the PST.

**Table 2:** The Psychological Skills Training Program.

Session (Week)	Program
1	About ADHD
2	The Importance of physical activity
3	Routines Setting
4	Goal Setting
5	Anxiety Control Training
6	Positive Self-Suggestion
7	Attention Focus Training
8	Review of Sessions 2–7

### Outcome measures

The final analysis will include data collected from a total of 54 children and their primary caregivers, with 18 children and their respective primary caregivers assigned to each of the two intervention groups and the control group: Intervention Group 1 (n = 36: 18 children, 18 parents), Intervention Group 2 (n = 36: 18 children, 18 parents), and the Control Group (n = 36: 18 children, 18 parents). Data from children and their primary caregivers will be analyzed separately.

The examiner will conduct the assessments while being blinded to the group assignment. Cognitive assessments will be administered by a certified clinical psychologist to quantitatively evaluate participants’ cognitive functions. Additionally, the physical activity level will be collected using the ActiGraph, and this data will be used to monitor the provided level of physical activity. The following assessment tools will be used for children with ADHD and their parents. For cognitive assessment, the Korean-Wechsler Intelligence Scale for Children, Fifth Edition (K-WISC-V) and the Visual-Motor Integration, Sixth Edition (VMI-6) will be used. For emotional assessment, the Revised Children’s Manifest Anxiety Scale (RCMAS) and the Children’s Depression Inventory (CDI) will be utilized. For behavioral assessment, physical activity levels will be measured using the Global Physical Activity Questionnaire (GPAQ). ADHD symptoms, social skills, and sleep quality will be evaluated using the Swanson, Nolan, and Pelham Questionnaire (SNAP), the Social Skills Rating System-Parent (SSRS-P), and the Korean Version of the Pittsburgh Sleep Quality Index (PSQI-K).

For primary caregivers, the assessment tools will include the GPAQ, the Parenting Stress Index-Short Form (PSI-SF), the Beck Anxiety Inventory (BAI), the Beck Depression Inventory (BDI), and the Korean Version of the Pittsburgh Sleep Quality Index (K-PSQI).

All groups will undergo three assessments for all measures except for the K-WISC-V. Intervention Groups 1 and 2 will be assessed before the intervention, immediately after the intervention (three months after the first assessment), and three months post-intervention. The control group will be assessed at baseline, after three months, and after six months. The K-WISC-V will be administered to all groups before the intervention and six months post-intervention. Detailed information about the outcome measures can be found in Table 3.

**Table 3:** outcome measures.

Participants	Variable	Clinical assessment items
**Children**	**Cognitive**	Wechsler Intelligence Scale for Children: WISC
		Visual-Motor Integration, Sixth Edition: VMI-6
	**Emotional**	Revised Children’s Manifest Anxiety Scale: RCMAS
		Children’s Depression Inventory: CDI
	**Behavioral**	Swanson, Nolan and Pelham: SNAP
		Social Skills Rating System-Parent: SSRS-P
		Korean Version of the Pittsburgh Sleep Quality Index: PSQI-K
		Global Physical Activity Questionnaire: GPAQ
**Parents**	**Emotional**	Parenting Stress Index-short form: PSI-SF
		Beck’s Anxiety Inventory: BAI
		Beck’s Depression Inventory: BDI
	**Behavioral**	Korean Version of the Pittsburgh Sleep Quality Index: PSQI-K
		Global Physical Activity Questionnaire: GPAQ

### Statistical analysis

The normality of the primary variables will be assessed using the Shapiro-Wilk test, and homogeneity of variances will be evaluated using Levene’s test. If normality is satisfied, a repeated measures ANOVA will be performed to examine the main effects of time, group, and their interaction. Mauchly’s test for sphericity will be conducted, and if violated, the Greenhouse-Geisser correction will be applied. If the normality assumption is violated, non-parametric alternatives will be implemented. The Kruskal-Wallis test will be used for between-group comparisons at each time point, while the Friedman test will be applied for within-group repeated measures. To adjust for multiple comparisons, Bonferroni correction will be applied as needed to control for Type I error. If significant interaction effects are observed in the repeated measures analysis, post-hoc tests will be conducted. For parametric statistics, paired t-tests will be used to compare within-group differences over time, and independent t-tests will be performed to compare mean differences between groups at the same time points. For non-parametric statistics, the Mann-Whitney U test will be used to compare two groups at a specific time point, and the Wilcoxon signed-rank test will be employed to analyze within-group differences between two time points.

All data collected in this study will be analyzed using SPSS 26 statistical software. The five subscales and total score of the Wechsler assessment will be analyzed separately. Since multiple comparisons will be made, a multiple comparisons issue may arise. To address this, the Bonferroni correction will be applied, setting the significance level to 0.0167. For other variables, the significance level will be set to 0.05 for analysis.

## Discussion

This study will explore the effects of PA and PST on children with ADHD and their primary caregivers. We will focus on understanding how these factors influence the cognitive, emotional, and behavioral functions of the children, along with the emotional and behavioral well-being of their parents.

Numerous intervention studies have shown that children and adolescents with ADHD experience significant improvements in the core symptoms of ADHD through engaging in moderate to vigorous PA and behavioral interventions [12], which generally include aerobic and manipulative activities [6,7,34]. The World Health Organization, the Centers for Disease Control and Prevention (CDC), and the American Academy of Pediatrics recommend behavior therapy for children with ADHD, which includes parent training. This training focuses on creating routines, organizing tasks, managing distractions, and setting goals to improve the child’s functioning. Additionally, a combined approach involving both behavioral therapy and medication has been shown to lead to better outcomes in academic performance, parent-child relationships, and social skills compared to using medication alone [35].

The intervention program’s PA component was explicitly developed to incorporate moderate to high-intensity PA. The PST includes routines, goal setting, positive self-talk, anxiety management, maintaining attentional focus, understanding ADHD, and recognizing the significance of PA. However, children with ADHD may struggle with completing tasks and sustaining attention, which highlights the need for additional support, such as involving parents, to help address these difficulties. Interestingly, a previous study that categorized PA interventions into parent and peer participation groups found that the parent involvement group demonstrated more significant improvements in cognitive, emotional, and behavioral outcomes [33]. This finding highlights the importance of parental involvement in interventions aimed at improving the functioning of children with ADHD, along with the significance of parent training [33,36–40].

Raising a child with ADHD imposes significant stress on parents [41]. This stress can become chronic and long-lasting, significantly negatively affecting the parent-child relationship and emotional well-being [37]. Although parents need psychological or physical interventions to cope with their stress, anxiety, and depression, most interventions are conducted separately for parents and children [21]. Joint participation in PA may encourage ongoing involvement in interventions and improve their overall effectiveness. Studies that compare groups who engage in PA together with those who do not have shown statistically significant improvements in functioning. This suggests that participating together could be a more effective intervention method for parents and children. It allows parents to support their children’s needs without having to dedicate additional time. While improving children’s symptoms is crucial, it’s also important to address maternal symptoms simultaneously. This highlights the importance and rationale for conducting this study.

This study has several potential limitations. First, participant recruitment is restricted to a single region within the metropolitan area, which may limit the generalizability of the findings. Second, the sample size for each group is relatively small, potentially reducing the clinical applicability of the results.

Despite these limitations, this study is significant as it examines the emotional and psychological aspects of both children and parents while quantitatively assessing changes in children’s cognitive performance using the Wechsler test.
